# Galectin-1-Dependent Mitochondria Apoptosis Plays an Essential Role in the Potential Protein Targets of DBDCT-Induced Hepatotoxicity as Revealed by Quantitative Proteomic Analyses

**DOI:** 10.1155/2022/5176300

**Published:** 2022-02-01

**Authors:** Jiayu Song, Yuan Ren, Lihua Liu, Yixin Wang, Chuandao Shi, Xiaoqing Ji, Yunlan Li, Qingshan Li

**Affiliations:** ^1^School of Pharmaceutical Science, Shanxi Medical University, Taiyuan 030001, China; ^2^School of Public Health, Shaanxi University of Chinese Medicine, Xi'an 712046, China; ^3^Key Laboratory of Innovative Drug for the Treatment of Serious Diseases Basing on the Chronic Inflammation, Shanxi University of Chinese Medicine, Taiyuan 030001, China

## Abstract

Di-n-butyl-di-(4-chlorobenzohydroxamato) tin(IV) (DBDCT), a new patent agent, exhibited strong antitumor activity. In some cases, its activity was close to or even higher than cisplatin, a first-line clinical metallic agent. Similar to platinum compounds, it also showed toxicity. However, the effective targets and mechanisms for specific toxicity and biological activity are still unclear. In this study, proteomic analysis revealed that 146 proteins (98 upregulated and 48 downregulated) were differentially identified by label-free LC-MS/MS after DBDCT treatment. Meanwhile, network analysis of these differential proteins suggested that protein Galectin-1 (Gal-1) could regulate the apoptosis process (15 related proteins), which played an essential role in the potential targets of DBDCT-induced hepatotoxicity. Furthermore, it was demonstrated that DBDCT might promote ROS production, activate NF-*κ*B p65, inhibit Ras and p-ERK1/2 expressions, increase the level of Gal-1, subsequently upregulate the expressions of Bax, p53, Fas, and FasL, and downregulate the expression of Bcl-2. As a result of these modulations, caspase cascades were finally activated, which executed apoptosis in HL7702 liver cells. Correspondingly, NAC (inhibitor of ROS), PDTC (inhibitor of NF-*κ*B), EGF (ERK1/2 activator), and OTX008 (inhibitor of Gal-1) were found to reverse and abolish the DBDCT-associated cytotoxicity partially. In conclusion, Gal-1 might be the potential target for toxicity and biological activity. Moreover, the present study will lay the groundwork for future research about di-n-butyl-di-(4-chlorobenzohydroxamato) tin structure optimization and developing it into a new potential anticancer agent.

## 1. Introduction

In recent years, significant attention has been paid to organotin(IV) compounds since there were reports on the antitumor activities of dialkyltin(IV) derivatives [1]. However, their clinical application was hindered by the toxicity of antitumor organotin(IV) agents [2]. Recently, a new patent agent (no. ZL01135148), di-n-butyl-di-(4-chlorobenzohydroxamato) tin(IV) (DBDCT) with arylhydroxamate ligand synthesized by our research group exhibited vigorous antitumor activity against several human tumors, such as bladder carcinoma, immature granulocyte leukemia, gastric carcinoma, and henrietta carcinoma. Its activity, in some cases, was close to or even higher than clinical first-line anticancer cisplatin [3]. Nevertheless, similar to other organotin(IV) agents, it showed specific toxicity, especially hepatotoxicity. The acute toxicity testing observed that the liver coefficient and the plasma biochemical indexes alanine aminotransferase (ALT) and aspartate aminotransferase (AST) were changed notably in mice after treatment with DBDCT [4]. However, the hepatotoxic molecular mechanisms and toxicity targets remain unclear.

Proteomics technology, which provides comprehensive protein information, is a more powerful tool than conventional bioassays to investigate the toxicological mechanisms and molecular targets exposed to any chemical [5, 6]. In the present study, high-throughput label-free LC-MS/MS-based quantitative proteomic analysis was employed to investigate the alterations of proteomic profiles in rat liver following DBDCT treatment. Some vital proteins related to apoptosis or mitochondrial functions were significantly changed. Hence, we speculate that the DBDCT-induced apoptosis might be a reason for anticancer activity and hepatotoxicity.

Apoptosis is the vital pathway for programmed cell death and can be initiated by several apoptotic stimuli, including abnormal intracellular calcium concentrations (excitotoxicity), oxidative stress, trophic factor deprivation, and DNA damage [7]. Galectin 1 (Gal-1), a *β*-galactoside binding mammalian lectin of 14 kDa, is widely distributed in various tissues and performs a variety of biological functions [8]. Recent studies show that overexpression of Gal-1 significantly reduces cell proliferation and induces apoptosis, such as in T cells and colorectal cancer cells [9–11]. As well, Gal-1 could induce a proapoptotic signal pattern as indicated by induction of proapoptotic Bad, decreased antiapoptotic Bcl-2 expression, and increased Bcl-2 phosphorylation [12]. On the other hand, members of the galectin family have been identified as death-inducing ligands [13]. Recent studies have provided experimental evidence that targeting Gal-1 to glycosides on Fas can activate the apoptotic death-receptor pathway [14]. Now research suggested that Gal-1 might be closely related to DBDCT-induced apoptosis through the mitochondrial or death-receptor pathways. These findings will be helpful to gain comprehensive insights into the targeted mechanisms regarding DBDCT-induced liver toxicity and provide valuable data for the reasonable use of this drug.

## 2. Materials and Methods

### 2.1. Reagents

DBDCT was synthesized by Shanxi Medical University with purity over 99.0% by HPLC analysis, and its structure was confirmed by mass spectrum (MS), nuclear magnetic resonance (^1^HNMR, ^13^CNMR, and ^119^SnNMR), and infrared spectroscopy (IR). Cell culture medium, trypsin, and fetal bovine serum (FBS) were obtained from Invitrogen, USA. Specific antibodies to GAPDH, Gal-1, NF-*κ*B, ERK1/2, GTP-Hras, p53, Bax, Bcl-2, Fas, and FasL were purchased from Santa Cruz, USA. NAC, PDTC, EGF, and OTX008 were obtained from Enzo Life Sciences, USA. Other reagents used in this study were purchased from Sigma, USA.

### 2.2. Animals and Treatment

Wistar rats (200∼250 g), purchased from the Laboratory Animal Center of Shanxi Medical University (Taiyuan, Shanxi Province, China), were kept in a well-ventilated animal house under standard conditions (temperature 25 ± 2°C, humidity 75%, and light/dark cycle 12 h/12 h). The animals were divided into two groups (*n* = 3). In the control group, rats were given saline. The treatment group was given DBDCT (5.0 mg/kg) once daily via tail intravenous injections for two days. At the end of the experiment, all the animals were euthanized under pentobarbital anesthesia. The appropriately sized livers were prepared for histological examination. The residual livers of the rats were collected and stored at −80°C or frozen in liquid nitrogen for proteomics analysis. The animal experiments were carried out following the National Institutes of Health Guide for the Care and Use of Laboratory Animals and were approved by the Animal Ethics Committee of Shanxi Medical University.

### 2.3. Histological Evaluation of Liver Tissues

The liver tissue specimens were obtained and fixed in 10% formalin for 24 h. The specimens were dehydrated, embedded by paraffin, evaluated through hematoxylin and eosin (H&E) staining, and then examined using a microscope (Olympus Corporation, Tokyo, Japan).

### 2.4. Sample Fractionation

Livers of the control and treatment groups were suspended in pyrolysis liquid for 30 min on ice. The mixture was centrifuged at 20,000 rpm for 15 min at 4°C to remove solid impurities. For quantitative proteomics analysis, the samples were reduced with 20 mM dithiothreitol, alkylated with 50 mM iodoacetamide, and digested with sequencing-grade modified trypsin at 37°C for 24 h. The solution was filtered by centrifugation at 12,000 rpm for 95 min at 4°C in a 10 k Amicon Ultra-0.5 filter. Then, the samples were redissolved in 0.1% formic acid for Liquid Chromatography-Mass Spectrometry (LC-MS/MS) analysis.

### 2.5. LC-MS/MS Analysis

A Thermo Scientific Easy-nLC 1000 mass spectrometer (Bremen, Germany) was used to analyze the liver samples. Peptide separations from the trypsin digestion of proteins were conducted using C_18_ easy-spray columns with a 250 nL/min flow rate. A solvent composition was 2% acetonitrile and 0.1% formic acid in the three times distilled water, and B solvent composition was 0.1% formic acid in acetonitrile. The peptides were eluted with the following gradients: 3%–8% B for 10 min, 8%–10% B for 55 min, 10%–20% B for 30 min, 20%–30% B for 15 min, 30%–90% B for 20 min, and 90% B for 10 min. Mass spectrometry was accomplished in the data-dependent mode, using the following parameters: 2.3 kV spray voltage, 250°C capillary temperature, 300–1800 m/z for a full-scan mass range, and an energy of 27% high-energy for C-trap dissociation (HCD) spectra. Each sample was run three times.

### 2.6. Label-Free Protein Expression Data Processing

MaxQuant software (version 1.5) was used to perform the label-free qualitative and quantitative analyses of the peptide and protein identification. Proteins were identified based on search engines, using the following search parameters: 25 mmu for fragment ion tolerance, 10 ppm for precursor ion tolerance, a fixed modification of cysteine alkylation, partial modifications of methionine oxidation and asparagine and glutamine taking off the amination, two missed cleavage sites, and trypsin as the digestion enzyme. Quantitative data were obtained with a minimum of two peptides per protein.

All were functionally annotated according to molecular functions and cellular components by Gene Ontology (GO) analysis using DAVID 6.7 (https//david.abcc.ncifcrf.gov) to obtain an overview of differentially expressed proteins.

### 2.7. Cytotoxicity Test

HL7702 liver cells were obtained from the Shanghai Institute of Biochemistry and Cell Biology (Shanghai, China), cultured in RPIM 1640 supplemented with 10% FBS and 1% penicillin/streptomycin, and maintained under standard conditions (37°C and 5% CO_2_). The effects of OTX-008 (inhibitor of Gal-1) and DBDCT on HL7702 cells proliferation were assessed by MTT assay. Briefly, HL7702 cells were plated in 96-well culture plates (1 × 10^6^ cells/well). After 24 h incubation, the cells were treated with DBDCT alone for 24 h or induced by OTX008 (10 *μ*mol/L) for 3 h firstly and then treated with DBDCT for 24 h. At the end of each treatment, 10 *μ*L of MTT (5 mg/mL) was then added to each well, and the cells were incubated for 4 h. The blue formazan salts produced from the cells were dissolved by adding 100 mL of DMSO, and the absorbance was measured at 490 nm using a microplate reader (Model 680, Bio-Rad, USA). Cell growth inhibition rate was expressed as the optical density ratio of the difference between the control and the treatment groups from three independent experiments.

### 2.8. Cell Morphological Assessment

HL7702 liver cells were cultured in RPIM 1640 medium until mid-log phase and then treated with DBDCT alone for 24 h or induced by OTX008 (10 *μ*mol/L) for 3 h firstly then treated with DBDCT for 24 h. The morphologies of cells were monitored under a light microscopy (BDS200-PH Inverted Microscope, Chongqing Optec Instrument Co., Ltd., China) at 24 h.

### 2.9. Measurement of Intracellular ROS Generation

The generation of intracellular ROS was evaluated using 2,7-dichlorofluorescein diacetate (DCFH-DA). Cells were harvested after different concentrations of DBDCT treatment for 24 h and washed twice with PBS, suspended in PBS, and then incubated with 10 *μ*mol/L DCFH-DA at 37°C in the dark for 30 min. The fluorescence intensities were measured using flow cytometry (Becton Dickinson, BD Biosciences, CA, USA) at an excitation wavelength of 488 nm, and fluorescence emission was measured in channel FL-1 at 530 nm.

### 2.10. Flow Cytometric Analysis of Apoptosis

HL7702 liver cells were incubated in a 12-well plate at a density of 2 × 10^5^ cells per well. The control and DBDCT-treated HL7702 cells were collected, washed, and resuspended in 500 *μ*L binding buffer at a concentration of 1 × 10^6^ cells/mL according to the manufacturer's protocol. The mixture of 5 *μ*L of Annexin V-FITC and 5 *μ*L of PI was added and incubated in the dark for 15 min. The samples were injected into the flow cytometer within 1 h.

### 2.11. RNA Extraction and Fluorescence Quantitative PCR Analysis

According to the manufacturer's instructions, total RNAs of HL7702 liver cells under different administration modes were isolated by TRIzol reagent and reverse-transcribed into cDNAs. The synthesized cDNA was amplified by quantitative real-time PCR using the SYBR kits. The reaction mixture was treated at 95°C for 30 s, followed by 40 cycles of 95°C for 5 s and 60°C for 30 s. Data from the reaction were collected and analyzed by complementary computer software. The relative level of the target gene was calculated by the 2^−ΔΔCT^ method and normalized to *β*-actin in each sample. The primers are shown in Table 1.

### 2.12. Caspase-3, -8, and -9 Proteases' Activities

Caspase-3, -8, and -9 activities were examined according to the manufacturer's instructions of caspase-3, -8, and -9 colorimetric assay kits. HL7702 cells were treated with DBDCT (3 *μ*mol/L) alone for 24 h or induced by OTX008 (10 *μ*mol/L) for 3 h firstly and then treated with or without DBDCT (3 *μ*mol/L) for 24 h. At the end of the treatment, the cells were collected by 0.25% trypsinization, washed with PBS, lysed with 100 mL of lysis buffer, and centrifuged for 15 min at 12000 rpm. Then, the supernatant (lysed protein) was transferred to a chilled fresh tube. The protein was adjusted to a concentration of 1–3 *μ*g/*μ*L by the Bradford method. Finally, 5 *μ*L of the Ac-DEVD-PNA (caspase-3)/Ac-IETD-PNA (caspase-8)/Ac-LEHD-pNA (caspase-9) was added to the protein samples, respectively, and incubated at 37°C for 2 h. Samples were monitored at 405 nm in the microplate reader.

### 2.13. Protein Extraction and Western Blot Analysis

HL7702 cells were harvested and lysed with RIPA lysis buffer after different treatments. The cells were disrupted and centrifuged at 12,000 rpm for 15 min at 4°C. Then, protein concentrations were quantitated using the BCA protein assay kits. Subsequently, the DBDCT-treated and control protein samples (25 *μ*g) were separated on a sodium dodecyl sulphate-polyacrylamide gel (SDS-PAGE) and then transferred onto nitrocellulose (NC) membranes. Then, the membranes were blocked in Tris-buffered saline-Tween 20 (TBST) containing 5% bovine serum albumin (BSA) for 2 h at room temperature and reacted with relevant antibodies (Gal-1, p53, Bax, Bcl-2, Fas, FasL, ERK1/2, NF-*κ*B, and GTP-Hras) for 12 h at 4°C, and GAPDH was used as the internal control, followed by incubation with secondary antibodies for 1 h. Finally, the membranes were exposed in a Molecular Imager ChemiDoc XRS System, and the western blot data were analyzed by ImageJ software.

### 2.14. Statistical Analysis

All experiments were repeated at least three times. The data were expressed as mean ± SD and statistically analyzed by the SPSS 27.0 software. The Student's *t*-test for comparisons between two groups was completed, and statistical significances were defined as a *p* value less than 0.05 for all analyses.

## 3. Results

### 3.1. Histopathology

**Figure 1:**
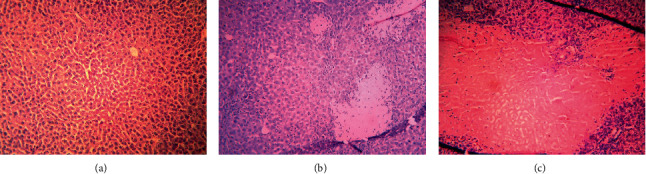
Changes in the hepatocytes stained H&E after DBDCT exposure (magnification: 10×). (a) H&E-stained image of the control hepatocytes. (b, c) The images of the hepatocytes treated with DBDCT.

Six rats were divided into 2 groups and administered with saline and DBDCT (5.0 mg/kg) by tail intravenous injections. After 2 days, the histopathological examination of liver tissues was performed to verify the liver toxicity of DBDCT. The histopathology of liver tissue was normal (Figure 1(a)) in the control group. But in the DBDCT-treated group, gross cellular damage was observed in the form of hepatic lesions, including focal necrosis (Figure 1(b)) and sheet necrosis (Figure 1(c)) of liver cells.

### 3.2. Identification of Differential Expression Proteins Induced by DBDCT Using LC-MS/MS and GO Analysis

**Figure 2:**
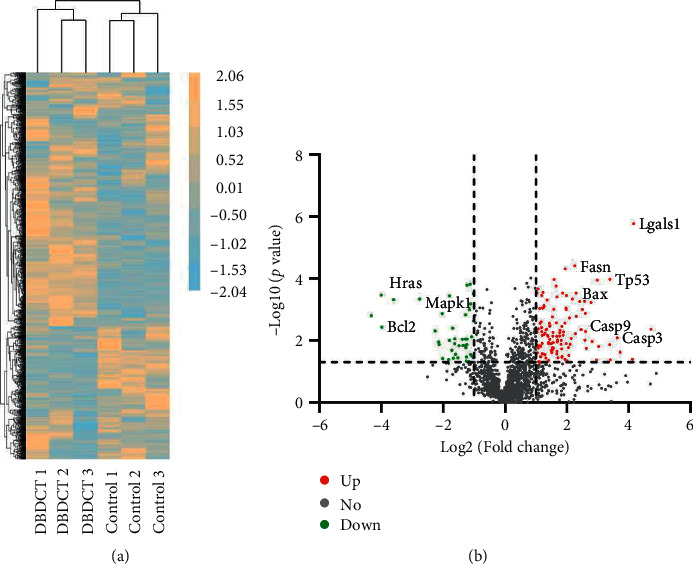
Proteomics analysis of DBDCT-treated rats (5.0 mg/kg). (a) Heat map of the significantly expressed proteins in the control and DBDCT groups. Orange and blue denote high and low expression, respectively. Proteins with similar expression patterns were clustered and are shown as the dendrogram. (b) Volcano plot of differentially expressed proteins in the control and DBDCT groups. Red dots show upregulated proteins, green dots show downregulated proteins, and grey dots show no statistically significant difference between the control and DBDCT groups.

Liver proteins were extracted and analyzed using label-free LC-MS/MS quantitative methods. In the control and DBDCT groups, a total of 2093 proteins were identified with a false discovery rate (FDR) of less than 1% at the peptide and protein level. According to the criteria of *p* value <0.05 and fold changes >2 or <0.5, 146 significant differential proteins were exhibited between the two groups. Of these, 48 proteins were downregulated and 98 proteins were upregulated in DBDCT-treated group compared with the control group. The differential expression proteins were presented in a global heat map (Figure 2(a)) and a volcano plot (Figure 2(b)).

**Figure 3:**
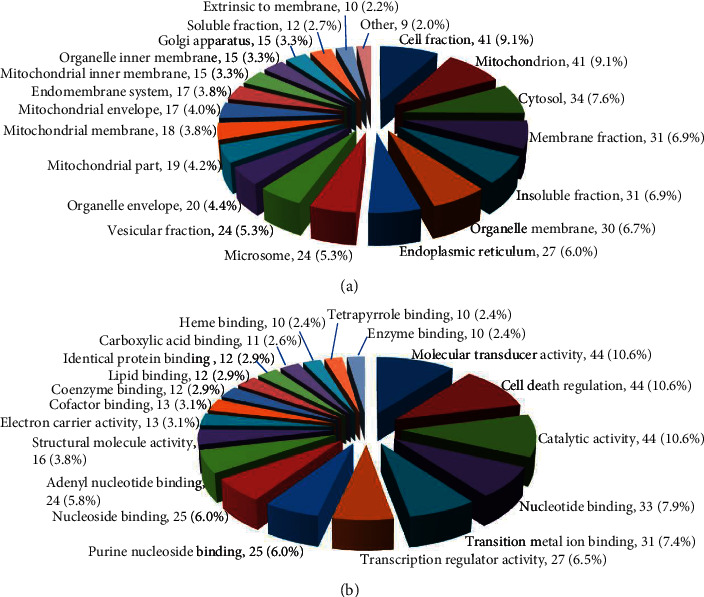
GO analysis of differential expression proteins. (a) Cellular component. (b) Molecular function.

To gain an insight into the biological significance, a total of 146 differentially expressed proteins were annotated by GO analysis and were classified into 20 significant GO terms in cellular component (Figure 3(a)) and 19 in molecular function (Figure 3(b)). Notably, 99 proteins were found at mitochondria, endoplasmic reticulum, or various membranes, which are the trigger sites or regulatory centers of apoptosis and 44 proteins were involved in the regulation of cell death. These findings indicated that the activities and hepatotoxic molecular mechanism of DBDCT might involve apoptosis.

### 3.3. Regulated Differentially Expressed Proteins Related to Apoptosis

**Figure 4:**
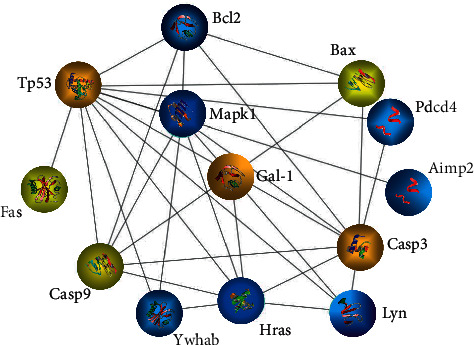
The PPI network of differentially expressed proteins was analyzed using the STRING database and the drawing tool Cytoscape. Yellow and blue denote high and low expressions, respectively.

According to the LC-MS/MS analysis and the UniProt database search, all of 15 proteins that are related to apoptosis in liver cells were identified successfully. Among these, 6 proteins were upregulated and 9 proteins were downregulated. A list of the identified proteins with their UniProt accession ID, protein name, gene name, fold change, and expression change was revealed in Table 2. Afterwards, the differentially expressed protein information from Table 2 was entered into the STRING database to analyze protein-protein interactions (PPI).  Figure 4 shows the PPI network generated by the drawing tool Cytoscape. As shown in Figure 4, Galectin-1 (Gal-1) is an essential node of the PPI network; thus, we speculate that Gal-1 protein may play a vital role in the effects of DBDCT.

### 3.4. High Expression of Gal-1 Induced by DBDCT

**Figure 5:**
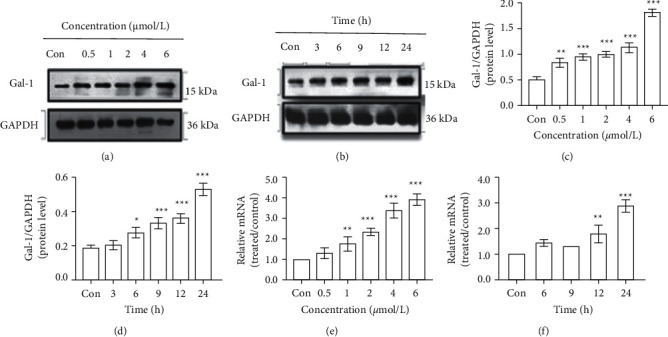
Effects of DBDCT on Gal-1 expression in HL7702 cells. (a) Immunoblotting results for 24 h. (b) Immunoblotting results with 2 *μ*mol/L DBDCT. (c) Relative protein level for 24 h. (d) Relative protein level with 2 *μ*mol/L DBDCT. (e) Relative mRNA level for 24 h. (f) Relative mRNA level with 2 *μ*mol/L DBDCT. Results were normalized according to GAPDH level. Each value represents the mean ± SD of three independent experiments. ^*∗*^*p* < 0.05, ^*∗∗*^*p* < 0.01, and ^*∗∗∗*^*p* < 0.001 compared with the control group.

As a highly conserved protein, Gal-1 participated in various biological functions, like cell proliferation, differentiation, and apoptosis. In this study, the expression of Gal-1 induced by DBDCT was evaluated by western blot and real-time PCR to verify the credibility of proteomic results. As shown in Figures 5(a) and 5(b), DBDCT treatment resulted in a dose- and time-dependent increase in Gal-1 protein expression level. Compared with the control group, different doses of DBDCT (0.5, 1, 2, 4, and 6 *μ*mol/L) for 24 h markedly increased Gal-1 protein level (Figure 5(c)). Furthermore, the Gal-1 protein level of HL7702 cells showed similar tendencies following exposure to 2 *μ*mol/L DBDCT at different periods (3, 6, 9, 12, and 24 h) (Figure 5(d)). As indicated by the real-time PCR results (Figures 5(e) and 5(f)), DBDCT also significantly upregulated the mRNA level of Gal-1 in a dose- and time-dependent manner. The above results were consistent with the proteomic results.

### 3.5. Regulated Effects of ROS/NF-*κ*B Pathway on the Expression of Gal-1 Induced by DBDCT

**Figure 6:**
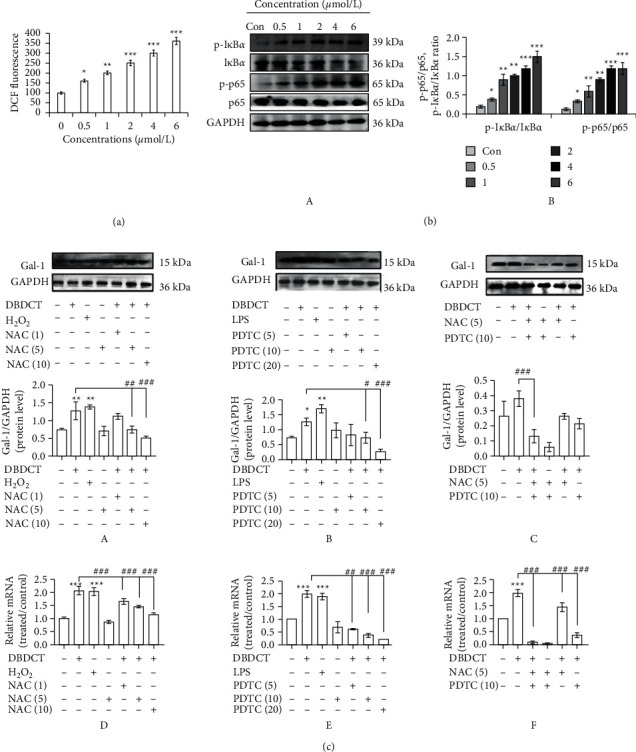
Effects of DBDCT on ROS generation and NF-*κ*B activation with different concentrations for 24 h and effects of NAC (inhibitor of ROS) and PDTC (inhibitor of NF-*κ*B) on the expression of Gal-1 induced by DBDCT in HL7702 cells. (a) ROS generation. (b) NF-kB activation. (c) Effects of inhibitor on the expression of Gal-1 induced by DBDCT (2 *μ*mol/L). (A) (D) Effects of NAC (1, 5, and 10 *μ*mol/L) on Gal-1 protein and mRNA level. (B) (E) Effects of PDTC (5, 10, and 20 *μ*mol/L) on Gal-1 protein and mRNA level. (C) (F) Effects of the combination of NAC (5 *μ*mol/L) and PDTC (10 *μ*mol/L) on Gal-1 protein and mRNA level. Results were normalized according to GAPDH level. Each value represents the mean ± SD of three independent experiments. ^*∗*^*p* < 0.05, ^*∗∗*^*p* < 0.01, and ^*∗∗∗*^*p* < 0.001 compared with the control. ^#^*p* < 0.05, ^##^*p* < 0.01, and ^###^*p* < 0.001 compared with the DBDCT alone.

Given that ROS/NF-*κ*B pathway may be related to the induction of apoptosis and mitochondrial dysfunction, a specific fluorescent dye DCFH-DA was used to explore whether DBDCT could stimulate ROS generation in HL7702 cells. As illustrated in Figure 6(a), the generation of intracellular ROS dramatically increased in DBDCT-treated groups with an apparent dose-effect relationship. Since a high level of ROS can activate NF-*κ*B, increase the chance of cell death, and inhibit cell growth, the expression levels of I*κ*B*α* and p65 with different DBDCT concentrations in HL7702 cells were measured. As shown in Figure 6(b), compared with the control group, the phosphorylation levels of I*κ*B*α* and p65 were increased in cells after treatment with DBDCT (0.5, 1, 2, 4, and 6 *μ*mol/L) for 24 h. It indicated that DBDCT might induce a significant accumulation of ROS and promote the activation of NF-*κ*B in HL7702 cells.

To examine whether the expression level of Gal-1 induced by DBDCT was affected by ROS or NF-*κ*B, we used the inhibitor of NF-*κ*B (PDTC) or ROS (NAC) with different concentrations to find the optimal effective concentration, respectively. Results of western blot analysis showed that compared with DBDCT alone, the combination of PDTC and DBDCT could partially reverse the upregulation of Gal-1 (Figure 6(c) (A)). Similar trends were detected by treatment with the combination of NAC and DBDCT (Figure 6(c) (B)). Furthermore, when treated with the combination of PDT, NAC, and DBDCT, the downregulation of Gal-1 protein expression was more significant (Figure 6(c) (C)). As illustrated in Figure 6(c) (D-F), the real-time PCR results were consistent with the above results. These findings suggested that ROS generation, NF-*κ*B activation, and the expressions of Gal-1 were affected by the intervening reagents in concentration-dependent manners, which indicated that NF-*κ*B and ROS might cooperatively regulate the expression of Gal-1 induced by DBDCT in HL7702 cells and Gal-1 might be identified as a possible potential target for toxicity and bioactivity.

### 3.6. Regulated Effects of Ras/ERK Pathway on the Expression of Gal-1 Induced by DBDCT

**Figure 7:**
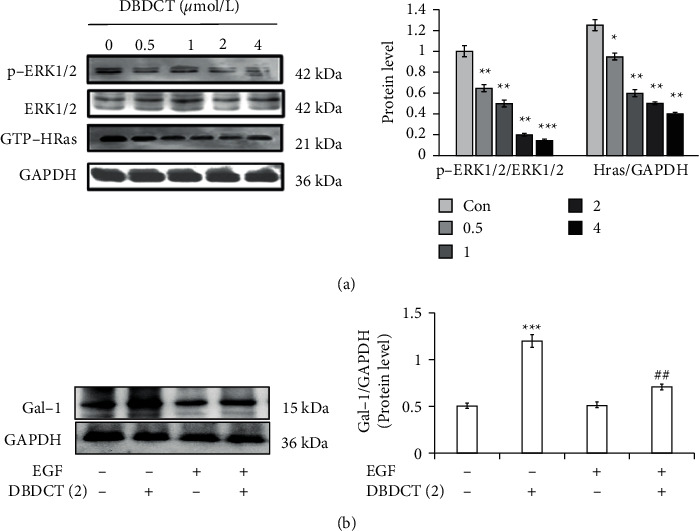
Effects of DBDCT on the expressions of p-ERK1/2, ERK1/2, and Ras with different concentrations for 24 h and effects of EGF (ERK1/2 activator) on the expressions of Gal-1 induced by DBDCT in HL7702 cells. (a) Expressions of p-ERK1/2, ERK1/2, and Ras. (b) Effects of EGF (30 ng/mL) on the expressions of Gal-1. Each value represents the mean ± SD of three independent experiments. ^*∗*^*p* < 0.05, ^*∗∗*^*p* < 0.01, and ^*∗∗∗*^*p* < 0.001, compared with the control. ^#^*p* < 0.05 and ^##^*p* < 0.01, compared with the DBDCT alone.

The Ras/ERK pathway, which negatively regulates various cellular responses, was assessed in HL7702 cells treated with different concentrations of DBDCT. As illustrated in Figure 7(a), compared with the control group, the levels of p-ERK1/2/ERK1/2 ratio and Ras were downregulated by 24 h of DBDCT-treatment in a concentration-dependent manner. In addition, to assess whether the Gal-1 protein level induced by DBDCT was affected by ERK1/2, the expression of Gal-1 protein in HL7702 cells treated with EGF (ERK1/2 activator) was examined. Western blot analysis showed that although the expression of Gal-1 protein was upregulated by treatment with DBDCT, the combination of EGF and DBDCT could partially abrogate the result (Figure 7(b)). Cumulatively, these results proposed that DBDCT might inhibit the ERK signalling pathway and subsequently affected the expression of Gal-1.

### 3.7. DBDCT-Induced Apoptosis Associated with Gal-1 in HL7702 Cells

**Figure 8:**
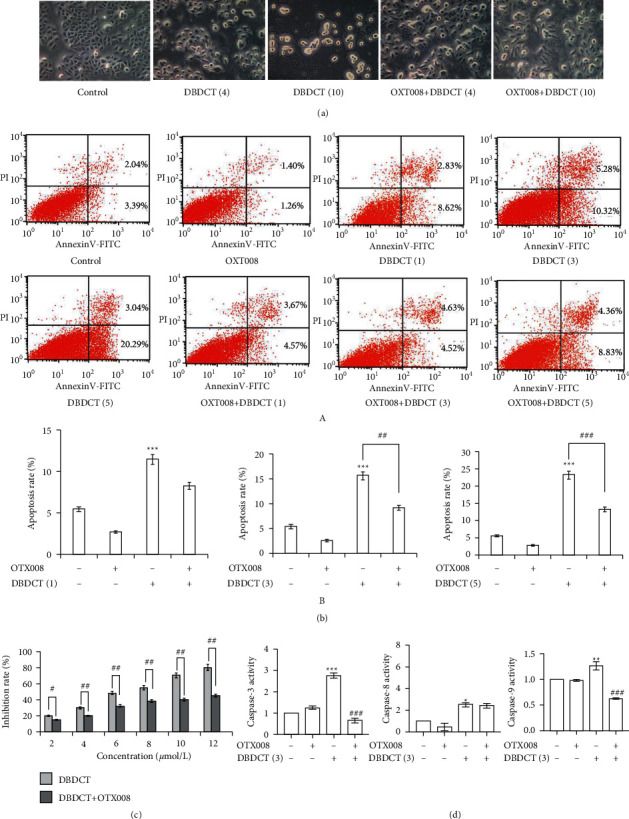
Effects of DBDCT on HL7702 cells proliferation and apoptosis, and the analysis of caspase-3, -8, and -9 activities. (a) Morphological changes of HL7702 cells by microscopic observation under an inverted light microscope (magnification: 20×). (b) Apoptosis of HL7702 cells. (A) The percentage of apoptotic cells was detected by flow cytometry using annexin V/PI double staining. (B) Apoptosis was assessed by counting the percentage of annexin V-positive cells. (c) Proliferation of HL7702 cells. (d) Analysis of caspase-3, -8, and -9 activities. HL7702 cells were treated with DBDCT alone for 24 h or induced by OTX008 (10 *μ*mol/L) for 3 h firstly to get a low Gal-1 level and then treated with DBDCT for 24 h. Each value represents the mean ± SD of three independent experiments. ^*∗∗∗*^*p* < 0.001 compared with the control group. ^#^*p* < 0.05, ^##^*p* < 0.01, and ^###^*p* < 0.001 compared with the DBDCT alone.

The differences in cell morphology were examined by light microscopy. The most conspicuous changes were observed in DBDCT (4 and 10 *μ*mol/L)-treated cells, including cell shrinkage and extensive detachment from the cell culture substratum (Figure 8(a)). But Gal-1 inhibitor OTX008 (10 *μ*mol/L for 3 h) could partially reverse and abolish the cytotoxic effects of DBDCT, indicating that Gal-1 might be identified as a possible potential target for toxicity and bioactivity. It will lay a better foundation for the future medicinal use of DBDCT. Meanwhile, MTT and flow cytometric analysis revealed that DBDCT promoted growth inhibition and cell apoptosis in a dose-dependent manner (Figures 8(b) and 8(c)), which was evident by the increased apoptotic ratio and growth inhibition rate, along with the increase in DBDCT concentration, while OTX008 pretreatment for 3 h could partially reverse the above results, which indicated that Gal-1 could be identified as a possible potential target for toxicity and bioactivity. To further validate the above results, a caspase activity assay kit was used. As shown in Figure 8(d), DBDCT increased the activities of caspase-3, -8, and -9 significantly. OTX008 pretreatment had little effect on DBDCT-induced activities of caspase-8, while it had a powerful inhibitory role in DBDCT-induced activities of caspase-3 and -9. So, we suspected that Gal-1 played a crucial role in Caspase cascades; meanwhile, it selectively participated in some apoptosis pathways induced by DBDCT.

### 3.8. DBDCT-Induced Apoptosis through Gal-1-Dependent Mitochondria Apoptosis Pathway

**Figure 9:**
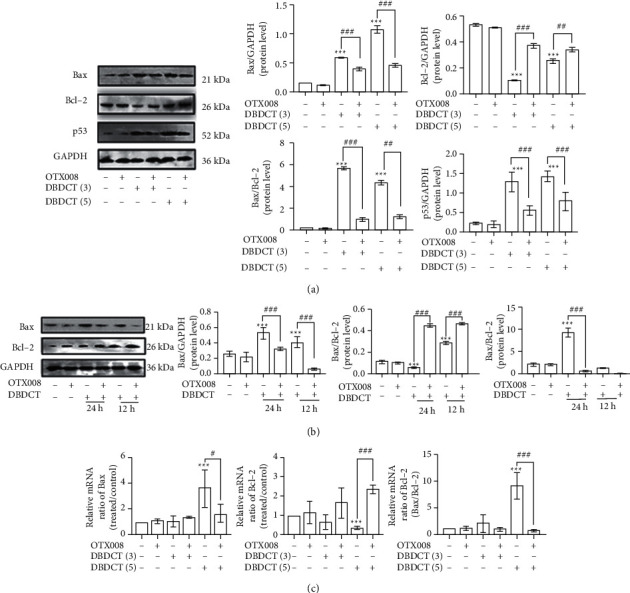
DBDCT-induced apoptosis via Gal-1-mediated pathway. (a) Western blot and relative protein levels of Bax, Bcl-2, and P53 with DBDCT at different concentrations for 24 h. (b) Western blot and relative protein levels of Bax and Bcl-2 with DBDCT (3 *μ*mol/L) for different time. (c) Relative mRNA levels of Bax and Bcl-2. HL7702 liver cells were treated with DBDCT or exposed to OTX008 for 3 h firstly and then treated with or without DBDCT. Each value represents the mean ± SD of three independent experiments. ^*∗∗∗*^*p* < 0.001 compared with the control. ^#^*p* < 0.05, ^##^*p* < 0.01, and ^###^*p* < 0.001 compared with the DBDCT alone.

The results of western blot analysis showed that compared with the control, treatment with increasing doses of DBDCT (3 and 5 *μ*mol/L) or for increasing durations (12 and 24 h) increased the Bax and p53 levels and decreased the Bcl-2 levels significantly, leading to a significant increase in the Bax/Bcl-2 ratio (Figures 9(a) and 9(b)). In contrast, when administered with OTX008 for 3 h before DBDCT treatment, the expression of Bax and p53 decreased whereas that of Bcl-2 increased, which accounted for the decreased ratio of Bax/Bcl-2 (Figures 9(a) and 9(b)) in HL7702 cells. Furthermore, as shown in Figure 9(c), the real-time PCR results were consistent with the western blot analysis. Accordingly, these findings suggested that Gal-1 might mediate DBDCT-induced apoptosis through changes in levels of Bax, p53, and Bcl-2, which are mitochondria-mediated apoptosis-related factors.

### 3.9. DBDCT-Induced Death Receptor Pathway Apoptosis Was Not Regulated through Gal-1-Dependent Way

**Figure 10:**
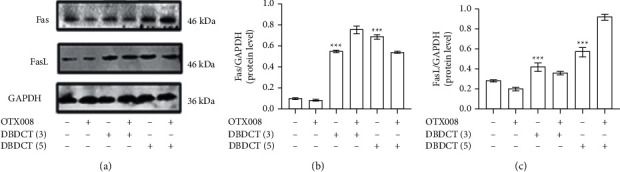
DBDCT-induced apoptosis via death receptor pathway. Western blot and relative protein levels of Fas and FasL. HL7702 cells were exposed to DBDCT at different concentrations (3 and 5 *μ*mol/L) for 24 h or exposed to OTX008 for 3 h firstly and then treated with or without DBDCT (3 and 5 *μ*mol/L) for 24 h. Each value represents the mean ± SD of three independent experiments. ^*∗∗∗*^*p* < 0.001 compared with the control.

Compared with the control, treatment of HL7702 cells with increasing doses of DBDCT (3 and 5 *μ*mol/L) increased the Fas and FasL levels significantly, while OTX008 pretreatment had little effect on the results (as shown in Figure 10). These results suggested that Gal-1 did not participate in the death receptor pathway, which might be mediated by DBDCT directly, but not through the Gal-1-dependent way, which was consistent with our previous speculation.

## 4. Discussion

DBDCT, one of the diorganotin(IV) arylhydroxamates synthesized by our group, showed prominent antitumor activity [15–17]. In this study, a histological evaluation was performed to confirm the hepatotoxicity in the rats injected with DBDCT. The results revealed that flake and focal necrosis were obvious in the DBDCT-treated rat livers. However, its hepatotoxicity molecular mechanisms and targets remained unclear. Nowadays, proteomics approaches play an important role in exploring drug targets and mechanisms. In this study, LC-MS/MS and label-free quantitative methods were used to screen for differentially expressed proteins in rat liver. About 49 proteins were 2 times downregulated, and 98 proteins were 2 times upregulated after treatment with DBDCT [18]. Meanwhile, among fifteen proteins involved in the cell apoptotic process or mitochondrial functions, Gal-1 might play a central role in the effects of DBDCT. Hence, we speculated that the hepatotoxicity of DBDCT might be associated with Gal-1-mediated apoptosis. Then, cellular and molecular experiments were carried out in HL7702 liver cells to validate the proteomic results and our conjecture.

Apoptosis is one type of cell death with a series of regulated signal cascades, in which Gal-1 plays an essential role in apoptotic signalling [19]. As the earliest and most relatively galectin family member, Gal-1 participates in various biological functions [20–22]. In the current study, it was demonstrated that DBDCT treatment increased the expression of Gal-1 at both protein and mRNA levels, which was consistent with the proteomic results. Meanwhile, we found that, besides inhibiting the proliferation of HL7702 cells, DBDCT showed an ability to trigger apoptosis in a dose-dependent manner. However, the increased apoptotic ratios induced by DBDCT could be reversed by Gal-1 inhibitor, confirming the occurrence of Gal-1-mediated programmed cell death.

It is well known that apoptosis is mediated by some initiator and executioner caspases and occurs via the mitochondria-mediated or cell death receptor-mediated pathway [23]. Activation of initiator caspase-9 mediates the mitochondria-mediated pathway. Alternatively, activation of the initiator caspase-8 mediates the cell death receptor-mediated pathway. Both initiator caspases converge onto a common pathway of executioner caspase-3 [24]. In this experiment, DBDCT could significantly activate caspase-3, -8, and -9; interestingly, gal-1 inhibitor pretreatment had little effect on caspase-8, while it had a decisive inhibitory role in caspase-3 and -9. Therefore, it seems that DBDCT induced apoptosis of HL7702 cells via the mitochondria-mediated and death receptor-mediated pathways, but Gal-1 might only selectively participate in the mitochondria-mediated pathway.

ROS/NF-*κ*B pathway is a redox signal pathway regulating the integrity of DNA repair systems, gene transcription, and translation under certain conditions. A high level of ROS can damage cellular components, induce cell cycle arrest, and, finally, cause cell death [25]. The current study found that DBDCT treatment could significantly increase ROS production and activate NF-*κ*B p65 compared with the control. Together, our prior studies had noted the importance of oxidative stress in DBDCT-mediated hepatotoxicity [17]. Moreover, given that the expression of Gal-1 is related to the ROS-dependent NF-*κ*B oxidative stress pathway [26–29], we assessed Gal-1 expression level in HL7702 cells treated with NF-*κ*B or ROS inhibitor. One interesting finding is that NF-*κ*B and ROS could cooperatively regulate the expression of Gal-1 induced by DBDCT (Figure 11①), and Gal-1 might be associated with DBDCT induced oxidative damage, which is a common cause of apoptosis.

ERK1/2 is a critical member of the mitogen-activated protein kinase family [30]. As a control switch, Ras can activate the ERK1/2 cascade [31]. What is more, recent studies have identified that inhibition of the Ras/ERK1/2 pathway, which plays a vital role in Gal-1 expression, can activate apoptosis pathways [32–37]. In the present study, the p-ERK1/2/ERK1/2 ratio and Ras levels were downregulated by DBDCT-treatment; meanwhile, the level of Gal-1 was decreased by the combination of ERK1/2 activator and DBDCT, although DBDCT itself did significantly increase the Gal-1 expression in HL7702 cells. It can thus be suggested that DBDCT could inhibit the ERK signalling pathway, which regulated the level of Gal-1 (Figure 11②).

To further validate whether DBDCT induced apoptosis of HL7702 cells via the Gal-1-dependent pathway, the expressions of apoptosis-associated proteins in HL7702 cells were detected. The expressions of the antiapoptotic protein Bcl-2 and the proapoptotic protein Bax and p53 are crucial determinants of the apoptotic response mediated by many agents [38, 39]. Accumulating evidence has shown that a decrease in the ratio of Bax/Bcl-2 inhibits Bax translocation to the mitochondria and then protects cells against apoptotic insults; however, a shift in the balance towards an excess of Bax evokes apoptosis [38, 39]. The results of this study showed that DBDCT treatment in HL7702 cells increased the Bax/Bcl-2 ratio and p53 levels; however, Gal-1 inhibitor pretreatment could reverse the above results. An implication of this was the possibility that DBDCT-induced apoptosis might be mediated through the gal-1-dependant mitochondria pathway. Cumulatively, these results proposed that DBDCT could activate ROS/NF-*κ*B pathway, meanwhile, inhibit the ERK signalling pathway, then affect the expression of Gal-1 and subsequently change the levels of Bax and Bcl-2, which triggered apoptosis (Figure 11①②).

The receptor-mediated pathway is triggered by the nerve growth factor/tumor necrosis factor (TNF) receptor superfamily (e.g., Fas). Upon binding with specific ligands (e.g., FasL), Fas initiates signalling pathways that culminate in apoptosis [25]. In this study, one interesting finding was that DBDCT induced the upregulation of the receptor pathway-related proteins, which were not significantly affected by pretreatment with Gal-1 inhibitor. Therefore, it could be assumed that Gal-1 might not mediate receptor pathway activation induced by DBDCT (Figure 11③).

Taken together, the results presented that DBDCT, a new organotin(IV) patented agent, has certain hepatotoxicity by inducing Gal-1-dependent apoptosis. Based on the clues provided by our proteomics and bioinformatics analyses, we are trying to identify the biological targets and toxic mechanisms of DBDCT. Despite these findings, this study has some limitations. Further investigations of whether DBDCT has a direct effect on Gal-1 are crucial for establishing underlying mechanisms.

## 5. Conclusions

**Figure 11:**
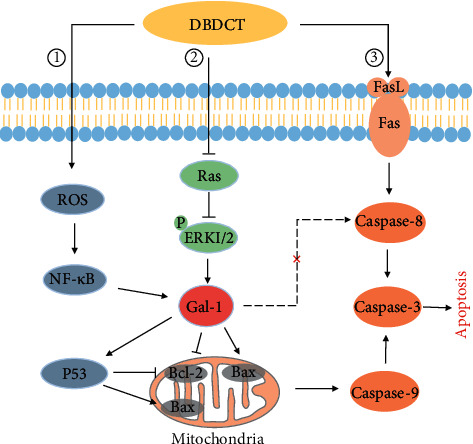
Schematic representation of possible mechanisms of DBDCT-induced apoptosis in HL7702 liver cells. ①② Gal-1-dependent mitochondria pathway. ③ DBDCT-regulated directly death receptor pathway which was not through Gal-1-dependent way (⟶ activation, ⊥ inhibition).

In conclusion, this study demonstrated that DBDCT, an antitumor diorganotin(IV) arylhydroxamate, could increase the expression of Gal-1, which was regulated by NF-*κ*B, ROS, and ERK1/2. In addition, our study also confirmed that DBDCT-induced apoptosis was related to the activation of the Gal-1-dependent mitochondria pathway and Gal-1-non-dependent receptor-mediated pathway, which was associated with increased levels of Bax and P53, decreased levels of Bcl-2, as well as with the activation of Fas/FasL and caspase cascades. These mechanisms have been summarized in Figure 11.

## Figures and Tables

**Table 1:** Nucleotide sequences of the primers.

Gene	Forward primer (5′-3′)	Reverse primer (5′-3′)	Product size (bp)
Bax	TGCTTCAGGGTTTCATCCAGGA	ACGGCGGCAATCATCCTCTG	172
Bcl-2	CTTCGCCGAGATGTCCAGCCA	CGCTCTCCACACACATGACCC	152
Gal-1	GGCAAAGACAGCAACAACCT	GGCCACACATTTGATCTTGA	94
*β*-Actin	CTACAATGAGCTGCGTGTGGC	CAGGTCCAGACGCAGGATGGC	270

**Table 2:** Regulated proteins related to apoptosis identified by LC-MS/MS and UniProt database.

Protein ID	Protein name	Gene name	Fold change	Expression change
P11762^a^	Galectin-1	Lgals1	17.96	Increase
P10361^a^	Cellular tumor antigen p53	Tp53	10.51	Increase
Q63690^a^	Apoptosis regulator BAX	Bax	4.93	Increase
Q63199^a^	Tumor necrosis factor receptor superfamily member 6	Fas	4.80	Increase
P55213^a^	Caspase-3	Casp3	12.39	Increase
Q920G4^a^	Caspase-9	Casp9	6.06	Increase
P63086	Mitogen-activated protein kinase 1	Mapk1	0.14	Decrease
P20171^a^	GTPase HRas	Hras	0.08	Decrease
Q32PX2	Aminoacyl tRNA synthase complex-interacting multifunctional protein 2	Aimp2	0.46	Decrease
Q9JLH7	CDK5 regulatory subunit-associated protein 3	Cdk5rap3	0.41	Decrease
P35213-2	14-3-3 protein beta/alpha	Ywhab	0.42	Decrease
Q07014-2	Tyrosine-protein kinase Lyn	Lyn	0.45	Decrease
P49950^a^	Apoptosis regulator Bcl-2	Bcl2	0.06	Decrease
Q4G009	Malignant T-cell-amplified sequence 1	Mcts1	0.32	Decrease
Q9JID1	Programmed cell death protein 4	Pdcd4	0.46	Decrease

*Note.* The differential proteins labeled ^a^ are the proteins that have been subsequently verified.

## Data Availability

Data are available upon request to the corresponding author.

## Supplementary Materials

Supplementary Materials One supplementary material is the Graphical abstract, and the other is the file about structural identifications of DBDCT.
