# Supplementary material for: Galectin-1-Dependent Mitochondria Apoptosis Plays an Essential Role in the Potential Protein Targets of DBDCT-Induced Hepatotoxicity as Revealed by Quantitative Proteomic Analyses
Source: Bioinorg Chem Appl. 2022 Feb 1;2022:5176300. doi: 10.1155/2022/5176300 (PMC8824758; doi:10.1155/2022/5176300)
Supplement: Supplementary Materials — One supplementary material is the Graphical abstract, and the other is the file about structural identifications of DBDCT. [file 5176300.f1.zip › 5176300.f1/structural identifications of DBDCT.pdf]

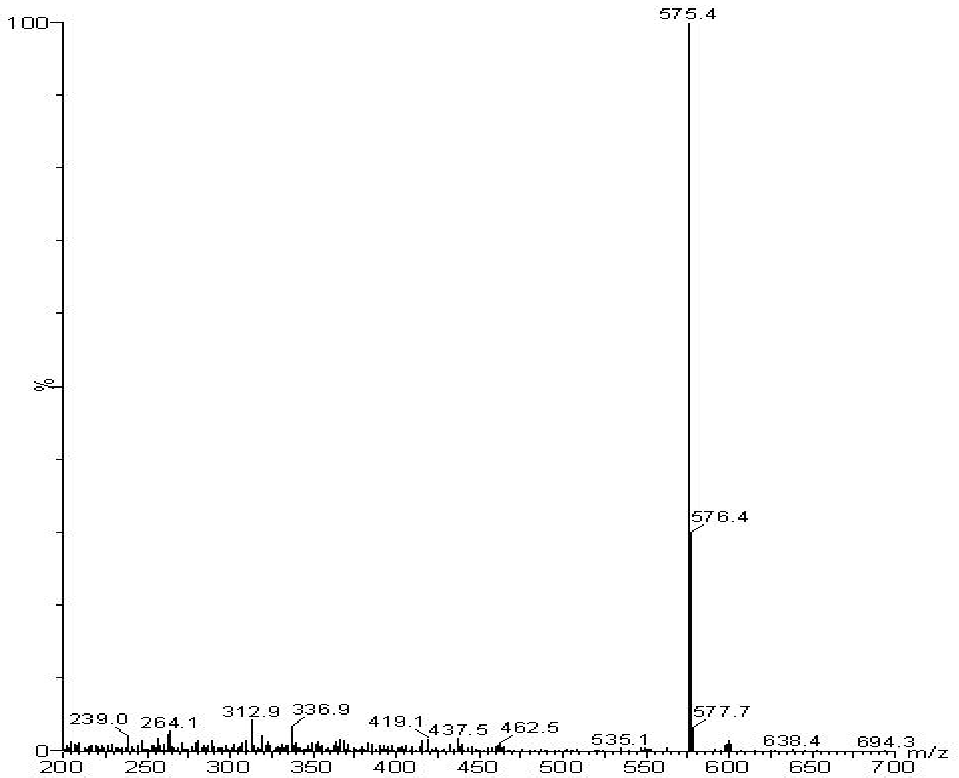

Figure 1 MS of **DBDCT**

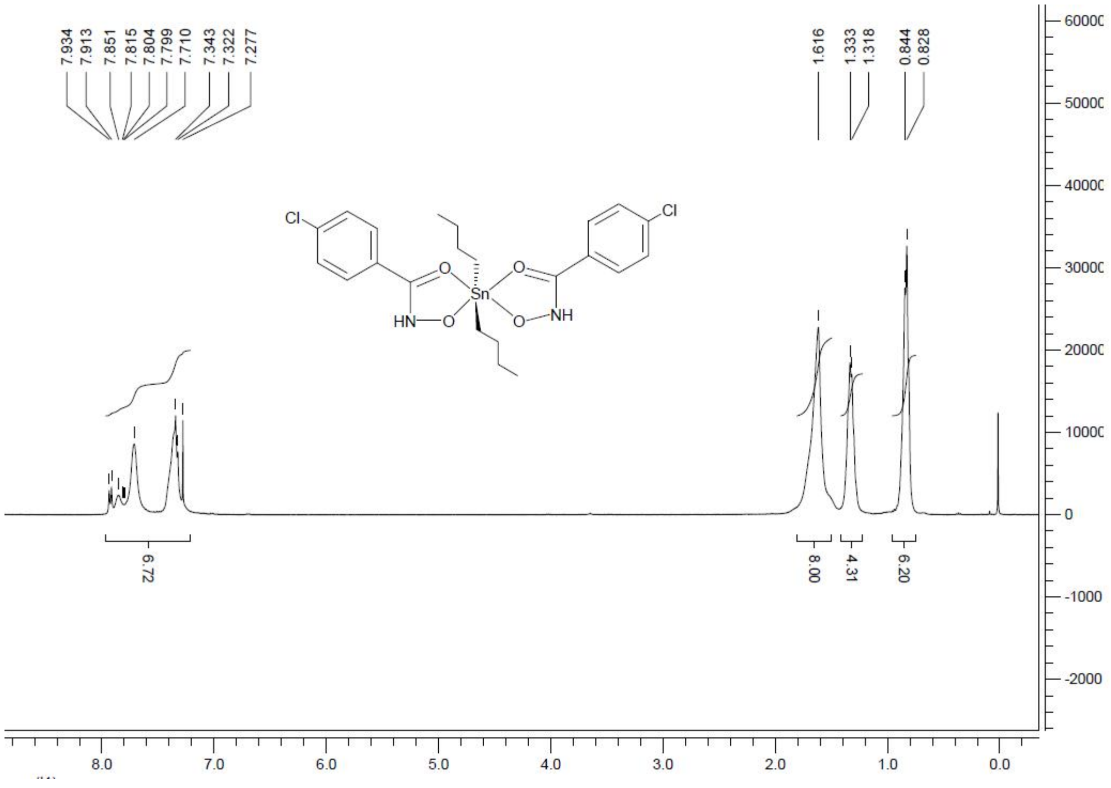

Figure 2 ^{1}HNMR of **DBDCT**

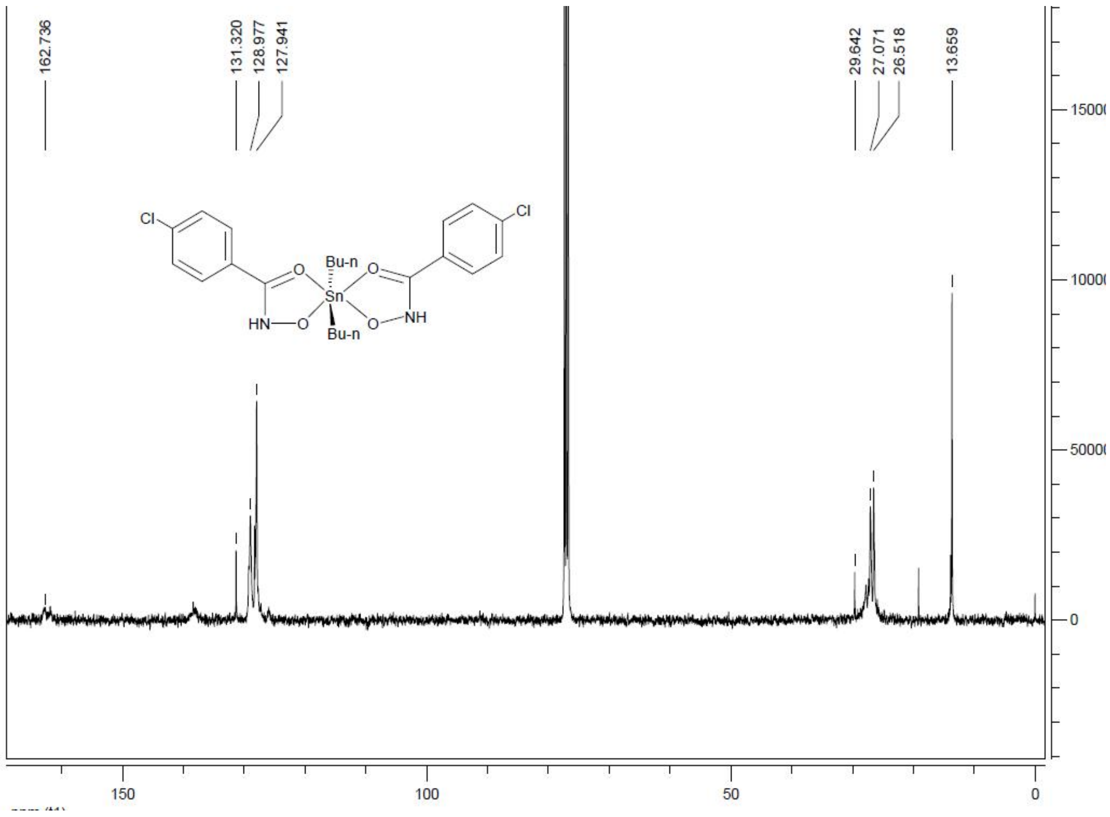

Figure 3 ^{13}CNMR of **DBDCT**

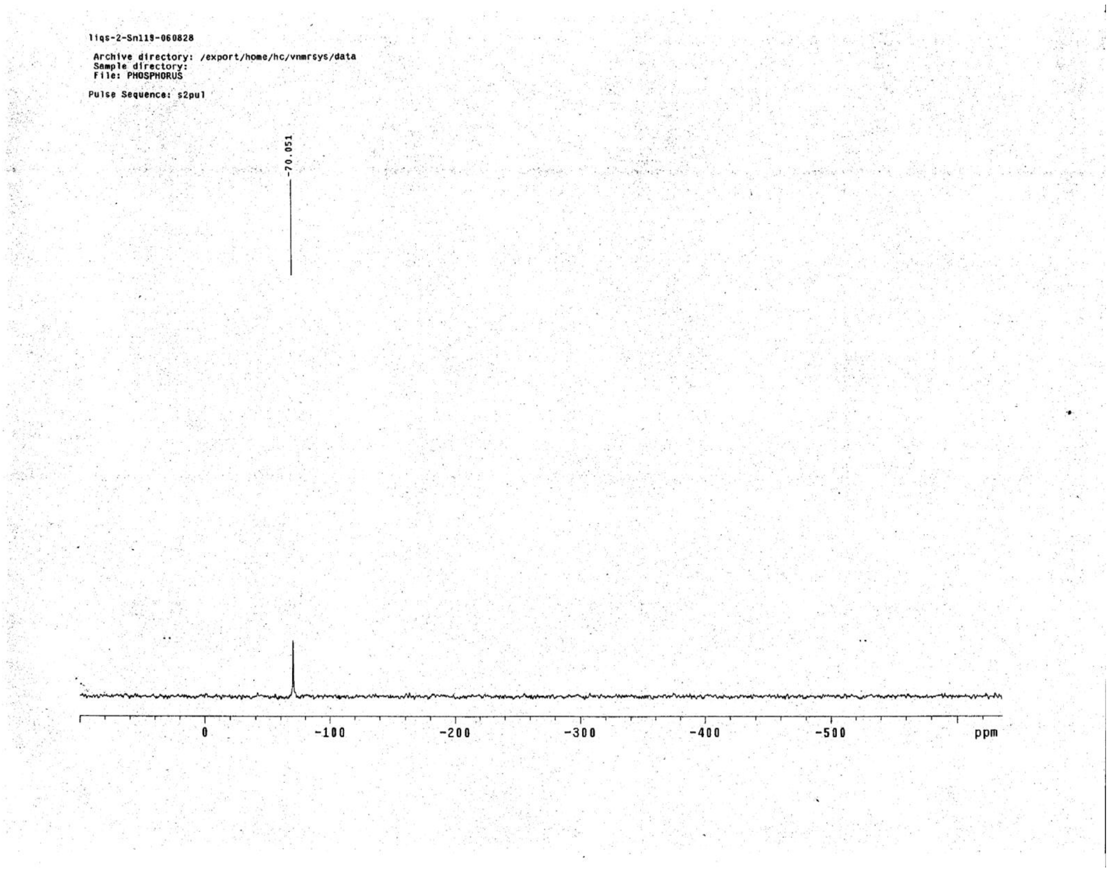

Figure 4 ^{119}SnNMR of **DBDCT**

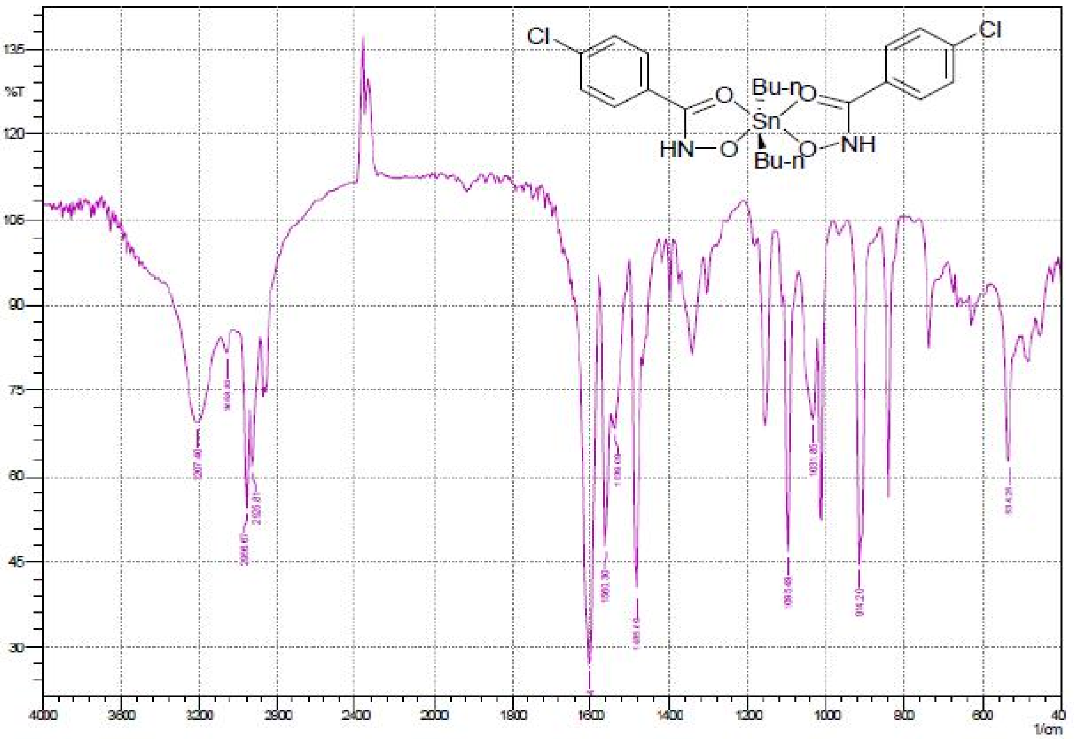

IR of DBDCT

Figure 5 IR of **DBDCT**
